# Macitentan, a double antagonist of endothelin receptors, efficiently impairs migration and microenvironmental survival signals in chronic lymphocytic leukemia

**DOI:** 10.18632/oncotarget.21341

**Published:** 2017-09-27

**Authors:** Rossana Maffei, Stefania Fiorcari, Tiziana Vaisitti, Silvia Martinelli, Stefania Benatti, Giulia Debbia, Davide Rossi, Patrizia Zucchini, Leonardo Potenza, Mario Luppi, Gianluca Gaidano, Silvia Deaglio, Roberto Marasca

**Affiliations:** ^1^ Division of Hematology, Department of Medical and Surgical Sciences, University of Modena and Reggio Emilia, Modena, Italy; ^2^ Department of Medical Sciences, University of Turin and Human Genetics Foundation, Turin, Italy; ^3^ Division of Hematology, Oncology Institute of Southern Switzerland and Institute of Oncology Research, Bellinzona, Switzerland; ^4^ Division of Hematology, Department of Clinical and Experimental Medicine, Amedeo Avogadro University of Eastern Piedmont, Novara, Italy; ^5^ Department of Oncology, Hematology and Respiratory Track Diseases, Azienda Ospedaliero - Universitaria Policlinico di Modena, Modena, Italy

**Keywords:** chronic lymphocytic leukemia, endothelin 1, macitentan, ibrutinib, microenvironment

## Abstract

The crosstalk between chronic lymphocytic leukemia (CLL) cells and tumor microenvironment is essential for leukemic clone maintenance, supporting CLL cells survival, proliferation and protection from drug-induced apoptosis. Over the past years, the role of several soluble factors involved in these processes has been studied. CLL cells express higher levels of endothelin 1 (ET-1) and ET_A_ receptor as compared to normal B cells. Upon ET-1 stimulation, CLL cells improve their survival and proliferation and reduce their sensitivity to the phosphoinositide-3-kinase δ inhibitor idelalisib and to fludarabine. Here, we demonstrate that CLL cells express not only ET_A_ receptor but also ET_B_ receptor. ET-1 acts as a homing factor supporting CLL cells migration and adhesion to microenvironmental cells. In addition, ET-1 stimulates a pro-angiogenic profile of CLL cells increasing VEGF expression through hypoxia-inducible factor-1 (HIF-1α) accumulation in CLL cells. Macitentan, a specific dual inhibitor of ET_A_ and ET_B_ receptors, targets CLL cells affecting leukemic cells migration and adhesion and overcoming the pro-survival and proliferation signals mediated by microenvironment. Furthermore, macitentan cooperates with ibrutinib inhibiting the BCR pathway and with ABT-199 disrupting BCL2 pathway. Our data describe the biological effects of a new drug, macitentan, able to counteract essential processes in CLL pathobiology as survival, migration, trafficking and drug resistance. These findings envision the possibility to interfere with ET receptors activity using macitentan as a possible novel therapeutic strategy for CLL patients.

## INTRODUCTION

Recirculation from blood to tissue compartments represents an essential multistep process in the pathophysiology of chronic lymphocytic leukemia (CLL) cells. CLL cells distribution into tissues is tightly regulated by the expression of adhesive molecules and chemokine receptors on leukemic cells, by the establishment of chemokine gradients and by physical contact with components of extracellular matrix or cellular elements of stromal and immune system. Inside tissues, invading CLL cells disrupt the physiological architecture of bone marrow (BM) and lymph nodes (LN), creating a favorable soil for maintenance and progression of leukemic clone [1]. CLL cells form areas of larger proliferating cells, known as pseudofollicles, establishing intimate contact with the accessory cells that favor CLL survival and proliferation, promote clonal evolution and protect cells from the effect of chemotherapeutics. In addition, CLL cells shape the physiologic network of microvessels in infiltrated-BM and LN compartments, by destabilizing the mature and quiescent status of blood vessels and promoting endothelial cells (EC) proliferation, migration and branching to form a dense weaving of functionally impaired microvessels [2]. In this context, CLL cells are not innocent bystanders, but actively dominate and model the surrounding microenvironment to aberrantly orchestrate the function of supporting elements and EC [3].

Endothelin 1 (ET-1) is a 21-aa peptide that mediates its action by activating two G-protein-coupled receptor (GPCR) subtypes, ET_A_ and ET_B_ receptors (ET_A_R and ET_B_R) [4]. Extracellular binding of ET-1 to ET receptors activates a non-linear, highly interconnected signaling network. Major pathways and effectors downstream of ET receptors include mitogen activated protein kinase (MAPK) and phosphatidylinositol 3-kinase (PI3K)/AKT signaling pathways, adenylyl cyclase and phospholipases (PLCβ and PLA2) [5]. In addition to its role as a potent endogenous vasoconstrictor and mediator of cardiovascular and renal disorders, aberrant activation of ET-1 axis is now recognized as a common mechanism underlying the progression of various solid tumors, including ovarian, prostate, colon, breast, bladder and lung cancer [4]. The ET-1 signaling exerts a pleiotropic action, as it can activate proliferation, confer apoptosis resistance, stimulate new blood vessel formation, modulate immune responses and promote invasion and metastatic dissemination [6, 7]. Endothelin receptors can be activated in cancer cells either through autocrine production of abnormal levels of ET-1 or through ligand secretion from microenvironmental stromal cells in a paracrine loop. Endothelin receptor blockade represents the most promising approach in controlling the pleiotropic activities of ET-1 [5, 6, 8]. Macitentan (*N*-[5-(4-Bromophenyl)-6-[2-[(5-bromo-2-pyrimidinyl)oxy]ethoxy]-4-pyrimidinyl]-*N*′-propylsulfamide) is a novel potent dual ET_A_/ET_B_ receptor antagonist as it inhibits [^125^I]-ET-1 binding to human recombinant ET_A_ receptors with an IC_50_ of 0.2 nM and to ET_B_ receptors with an IC_50_ of 391 nM. Treatment with macitentan leads to inhibition of growth, vascularization, intravasation, and metastatic dissemination, and also overcomes chemoresistance in solid tumors [9–13].

We previously demonstrated that CLL cells express higher levels of ET-1 as compared to normal B cells. ET-1 stimulation protects CLL cells from spontaneous apoptosis, stimulates proliferation and reduces leukemic cells sensitivity to the phosphoinositide-3-kinase δ inhibitor idelalisib and to fludarabine. Increased plasma levels of big ET-1, the precursor of ET-1, were detected in patients with unfavourable prognostic factors and shorter time to first treatment [14].

Here, we report that blocking ET receptors by macitentan hampers CLL cells migration, facilitates CLL cell death and overcomes survival advantage mediated by microenvironmental elements. Interestingly, macitentan interferes with B-cell receptor (BCR) activation and improves ABT-199 effects on CLL cells in contact with stromal supporting cells. We also demonstrate that macitentan impairs CLL cells proliferation, interferes with β-catenin signaling and reduces VEGF expression in CLL cells by decreasing hypoxia-inducible factor-1 (HIF-1α) accumulation. These findings envision the possibility to interfere with ET receptors activity using macitentan as a possible novel therapeutic strategy for CLL patients.

## RESULTS

### An increase in big ET-1 plasma levels accompanies CLL disease progression

**Figure 1:**
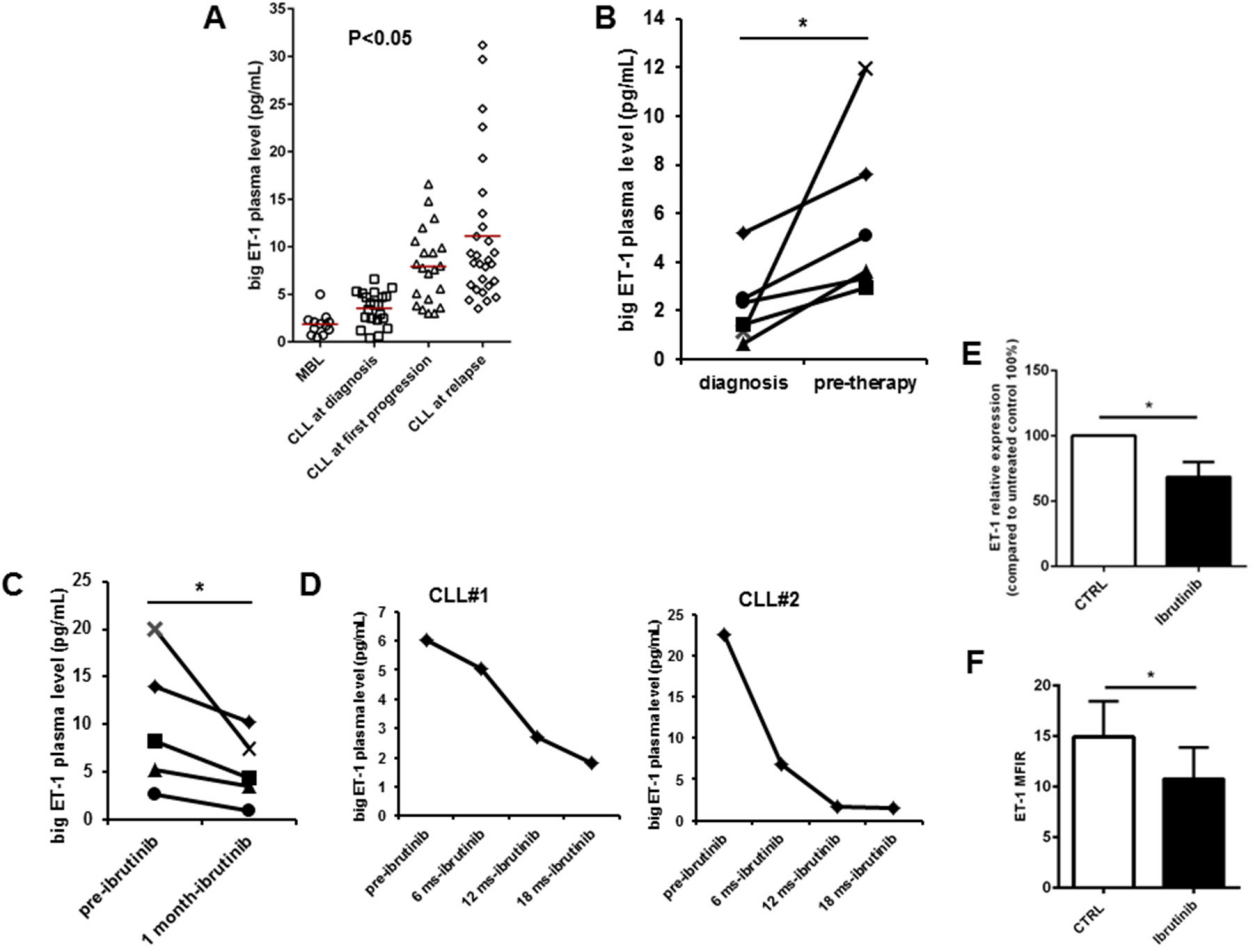
ET-1 plasma levels are associated with CLL disease progression **(A)** Dot plots depict the levels of big ET-1 in plasma samples collected from MBL (n=12), CLL at diagnosis (n=21), CLL at first progression (n=21) and CLL at relapse (n=28). **(B)** Measurement of big ET-1 levels in plasma samples longitudinally collected at diagnosis and pre-therapy in 6 patients. **(C)** Values of ET-1 plasma levels of pre-treatment and after one month of treatment with ibrutinib were evaluated in 5 different patients (n=5, p<0.05). **(D)** For two representative CLL patients, plasma samples were collected pre-treatment and after different time-points of ibrutinib treatment. As shown after long-term treatment with ibrutinib, a decrease of big ET-1 was measured. **(E** and **F)** Bar diagrams show transcriptional levels of ET-1 measured by quantitative reverse-transcription PCR and ET-1 protein levels quantified by flow cytometry (MFIR) in CD19+ CLL cells cultured *in vitro* in presence or absence of ibrutinib (n=6, p<0.05).

We previously reported that patients with higher levels of big ET-1 in plasma at diagnosis experience shorter time to first treatment [14]. Here, we asked whether ET-1 accumulation in plasma effectively accompanies CLL natural history prior to treatment and whether its level is affected by ibrutinib treatment. So, we evaluated big ET-1 levels in plasma samples collected from patients at different clinical time points, including 12 monoclonal B-cell lymphocytosis (MBL), 21 CLL at diagnosis, 21 CLL at progression prior to first treatment, and 28 CLL at relapse. In plasma samples of this cohort, big ET-1 was detectable at highly variable levels ranging from 0.5 to 31.2 pg/mL. We observed a significant increase in big ET-1 levels in CLL at diagnosis compared to MBL patients (3.5±0.4 pg/mL vs. 1.9±0.3 pg/mL, respectively, p=0.006, Figure 1A). Interestingly, big ET-1 concentration doubled in plasma of CLL at first progression compared to CLL at diagnosis (7.9±0.8 pg/mL vs. 3.5±0.4 pg/mL, respectively, p<0.0001). Accordingly, six patients with samples longitudinally collected at diagnosis and before first therapy showed increased levels of big ET-1 (Figure 1B). Furthermore, we detected an additional increase of big ET-1 level at relapse after 1 or multiple therapies (11.1±1.4 pg/mL). Big ET-1 plasma levels did not correlate with white blood cell (WBC) count seen in CLL patients (p=n.s.). In particular, two untreated CLL patients with multiple samples longitudinally collected for 4 and 8 years during follow-up were analyzed. Although experiencing a progressive slow increase in WBC count, big ET-1 plasma levels remain constantly low (Supplementary Figure 1). A representative CLL patient with multiple plasma samples collected at diagnosis and during 3-year follow-up before therapy showed a roughly similar WBC count but a huge increase in big ET-1 levels from 0.9 to 88 pg/mL (Supplementary Figure 1). These observations define ET-1 expression as a hallmark of CLL clone instead of a mere effect of CLL accumulation.

Lastly, we evaluated 5 patients receiving ibrutinib, finding a significant decrease of big ET-1 in plasma just after 1 month of treatment, despite the huge increase in lymphocytosis related to the treatment (p<0.05, Figure 1C). As shown in Figure 1D, two representative CLL patients with plasma samples collected after long-term treatment with ibrutinib (6, 12 and 18 months) confirmed the decrease of big ET-1. Accordingly, ET-1 mRNA and protein expression in CLL cells was reduced by *in vitro* treatment with ibrutinib (Figures 1E and 1F). Overall, these findings strongly suggest a correlation between ET-1 expression and CLL progression *in vivo*. Due to the pleiotropic action of ET-1 signaling in several tumor settings, we asked if ET-1 may be involved in aberrant functions leading to CLL progression and relapse, i.e. prolonged survival, proliferation, microenvironmental support and chemoprotection.

### Macitentan interferes with survival signals in CLL

**Figure 2:**
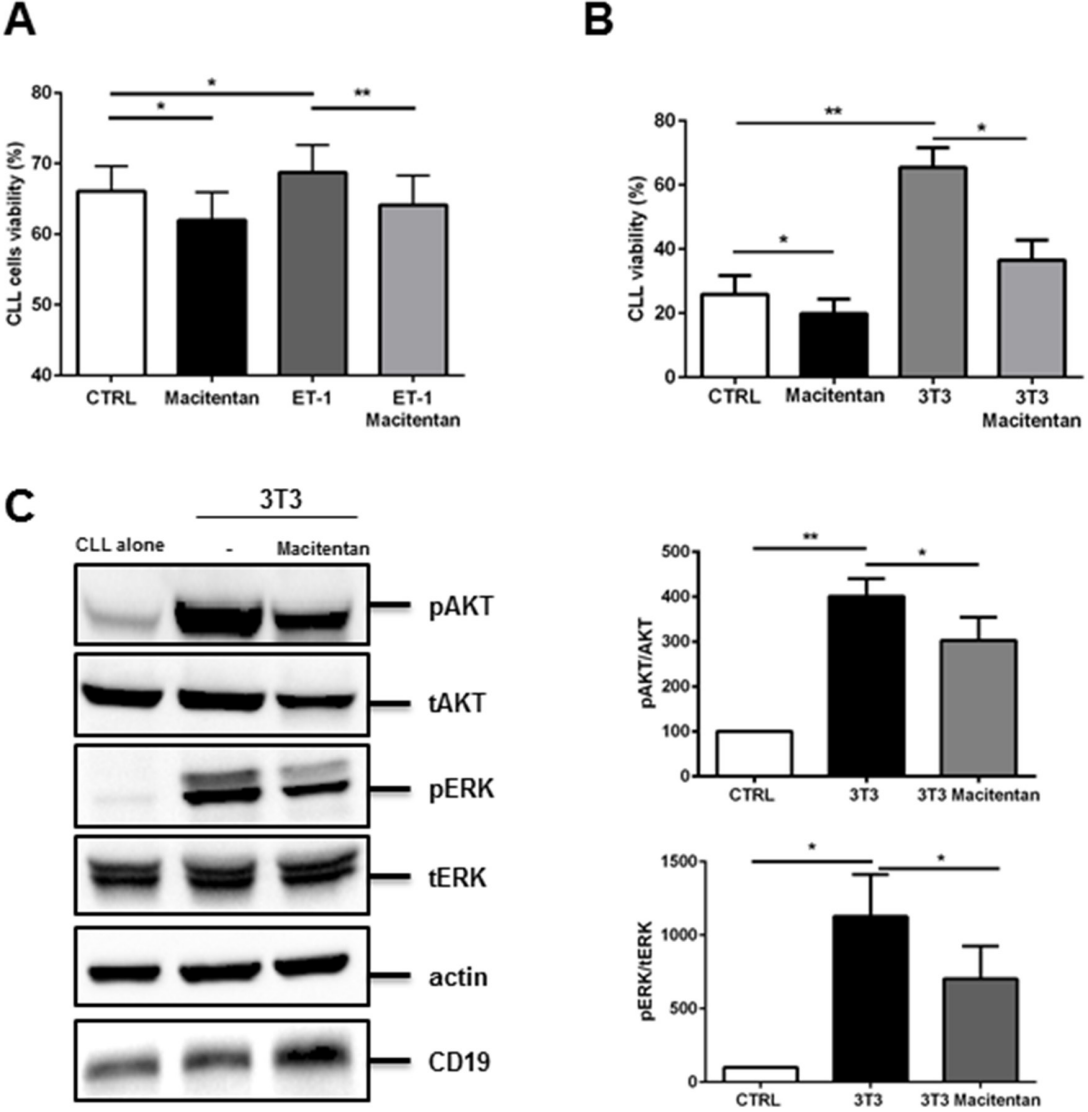
Macitentan interferes with survival signals in CLL cells **(A)** CLL cells were cultured with the addition of recombinant ET-1 peptide either in presence or absence of macitentan. Histograms represent mean±SEM at 24h of 6 CLL patients evaluated in 3 independent experiments (P^*^<0.05, ^**^P<0.01). **(B)** CLL cells (n=5), pretreated or not with macitentan, were cultured on 3T3 cell line layer for 96 hours. Bar diagrams represent data as mean±SEM of 5 CLL. **(C)** CD19+ CLL cells were cultured either alone or in contact with 3T3 in presence or absence of macitentan for 24 hours. Immunoblots show pAKT, pERK and the corresponding total protein, actin and CD19 in one representative CLL samples. Bar diagrams depict densitometric quantification of bands relative to pAKT/tot AKT, and pERK/tot ERK ratio of the corresponding condition, normalized on CD19 amount and β-actin. Data are presented as mean ± SEM of 5 different CLL samples (n=6, ^*^P<0.05; ^**^ P<0.01).

Here, we inspected CLL cells for the presence of ET_B_ receptor finding that leukemic cells effectively express both ET receptors (Supplementary Figure 2). This feature prompted us to evaluate the effects of macitentan, a novel non-peptide double antagonist of ET_A_R and ET_B_R, on CLL cells. After investigating the effects of the inhibitor on CLL viability, we found that macitentan slightly favoured spontaneous apoptosis and affected ET-1 mediated survival advantage of CLL cells (Figure 2A). Long-term survival of CLL cells is promoted by close contact with microenvironmental elements such as stromal cells. We wondered if macitentan may interfere with such mutualistic interactions, finding its ability to almost completely nullified stromal cell-mediated survival advantage after 4 days of co-culture (Figure 2B). We investigated the effect of macitentan on signaling pathways involved in this crosstalk and we found a strong induction of AKT and ERK phosphorylation in CLL cells cultured with 3T3 compared to control (n=6, p<0.01 and p<0.05). Moreover, in presence of macitentan a significant decrease of AKT and ERK phosphorylation was detected in the co-culture model (n=6, p<0.05, Figure 2C).

**Figure 3:**
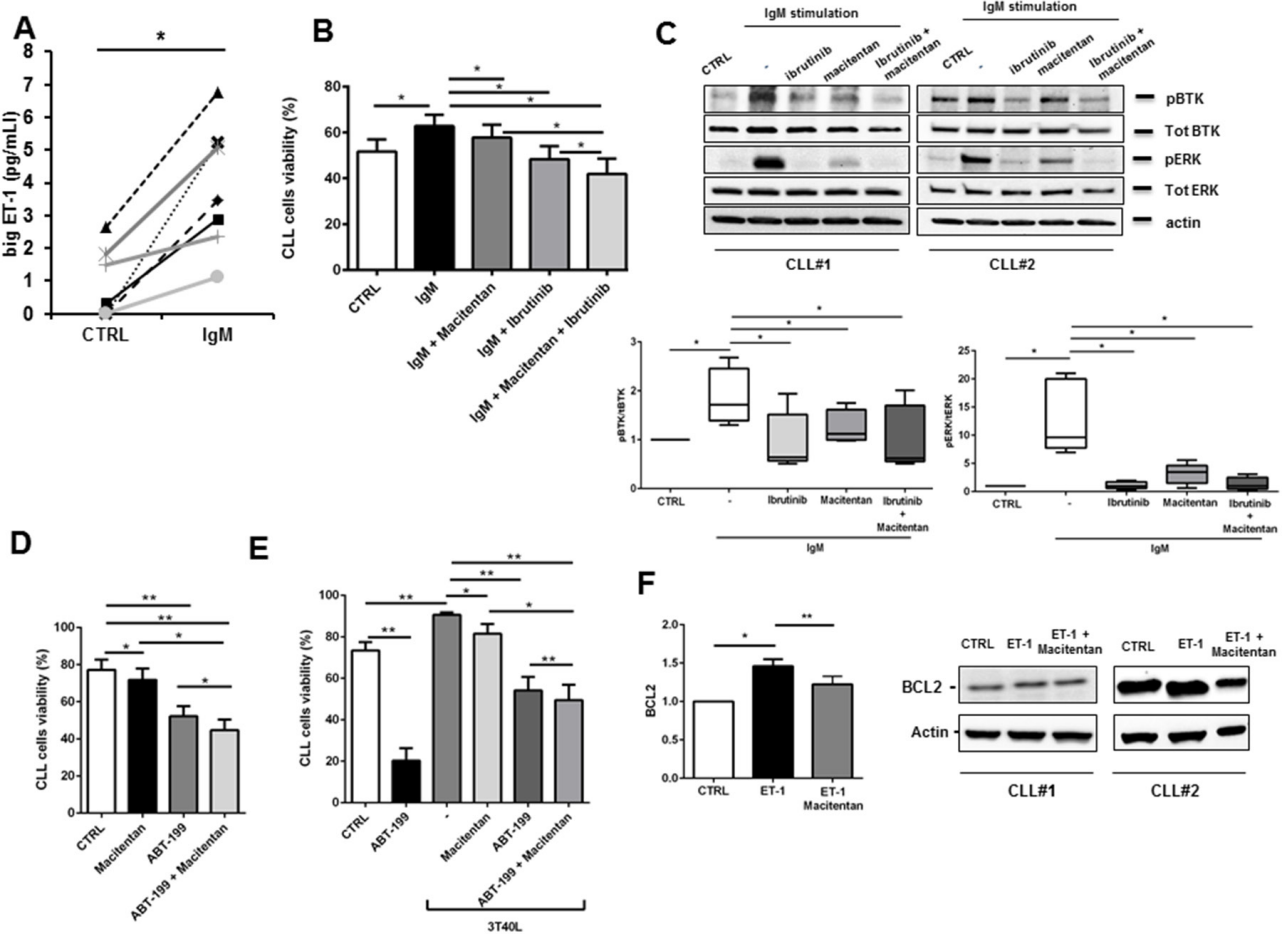
Macitentan blocks BCR signaling and promotes apoptosis in combination with ibrutinib and ABT-199 in CLL cells **(A)** Big ET-1 level was measured in conditioned medium collected from unstimulated CLL cells or stimulated with F(ab’)2 fragment to human IgM (n=6, P<0.05). **(B)** CLL cells were pre-treated with macitentan or ibrutinib or both for 1 hour and then stimulated with anti-IgM. CLL cells viability was measured after 24h, 48h and 72h. Bar diagrams show cell viability as mean±SEM at 48 hours of 5 CLL patients (^*^P<0.05). **(C)** Serum-starved CLL cells were pretreated or not with ibrutinib, or macitentan or combination of both for 1 hour before stimulation with anti-IgM for 10 minutes. The immunoblots depict BTK, ERK phosphorylation in two representative cases. Below, box plots show densitometric quantification of bands relative to pBTK/tot BTK and pERK/tot ERK normalized on β-actin (n=5, ^*^P<0.05). **(D)** CLL cells were pre-treated with macitentan for 1 hour and ABT-199 was added in culture. Bar diagrams show CLL cells viability measured after 24h of culture (n=6, ^*^P<0.05, ^**^P<0.01). **(E)** CLL cells were pre-treated with macitentan for 1 hour and then cultured onto 3T40L cell layer for 3 hours. Then, ABT-199 was added and CLL cells viability was measured after 24h, 48h and 72h. Bar diagrams show CLL cells viability as mean±SEM after 48 hours of culture (n=4, ^*^P<0.05, ^**^P<0.01). **(F)** Serum-starved CLL cells were either pre-treated or not with macitentan for 1 hour and then cultured for 24 hours with ET-1. On the left, bar diagram depicts densitometric quantification of bands relative to BCL-2 either in presence or absence of the corresponding treatment, normalized on β-actin. Data are presented as mean ± SEM of 3 different CLL samples (^*^P<0.05). On the right, two representative samples are depicted.

Beside cellular interplay, BCR activation represents a key driver for maintenance and progression of CLL cells. We stimulated BCR signaling on CLL cells by using a F(AB’)2 fragment to human IgM, observing a significant increase in ET-1 secretion by leukemic cells (Figure 3A). In addition, BCR triggering led to an increase in CLL survival that was partially hampered by macitentan alone through interference with BTK phosphorylation and ERK signaling pathway (Figures 3B-3C). The synergistic combination of macitentan with the BTK inhibitor ibrutinib completely nullified BCR-mediated survival and intracellular signaling, indicating the convergence of BCR and ET receptors downstream pathways in CLL cells (Figures 3B-3C).

A promising novel therapeutic strategy for CLL is to target the apoptotic machinery directly by the so-called BH3-mimetics, i.e. BCL2-specific compound ABT-199 or venetoclax. Interestingly, an additional synergistic reduction in CLL survival was detected in an overnight incubation when macitentan was used in combination to 0.001μM BCL2 inhibitor ABT-199 as compared to ABT-199 alone (Figure 3D). Moreover, a reduction in CLL resistance to 1μM ABT-199 mediated by contact with stromal cells expressing CD40L (3T40L) was also found with the addition of macitentan (Figure 3E). Accordingly, ET-1 stimulation increases BCL2 expression in CLL and treatment with macitentan prevents this unwanted effect that limits ABT-199 efficacy towards CLL cells (Figure 3F).

### Macitentan counteracts CLL proliferation by inhibiting β-catenin accumulation

**Figure 4:**
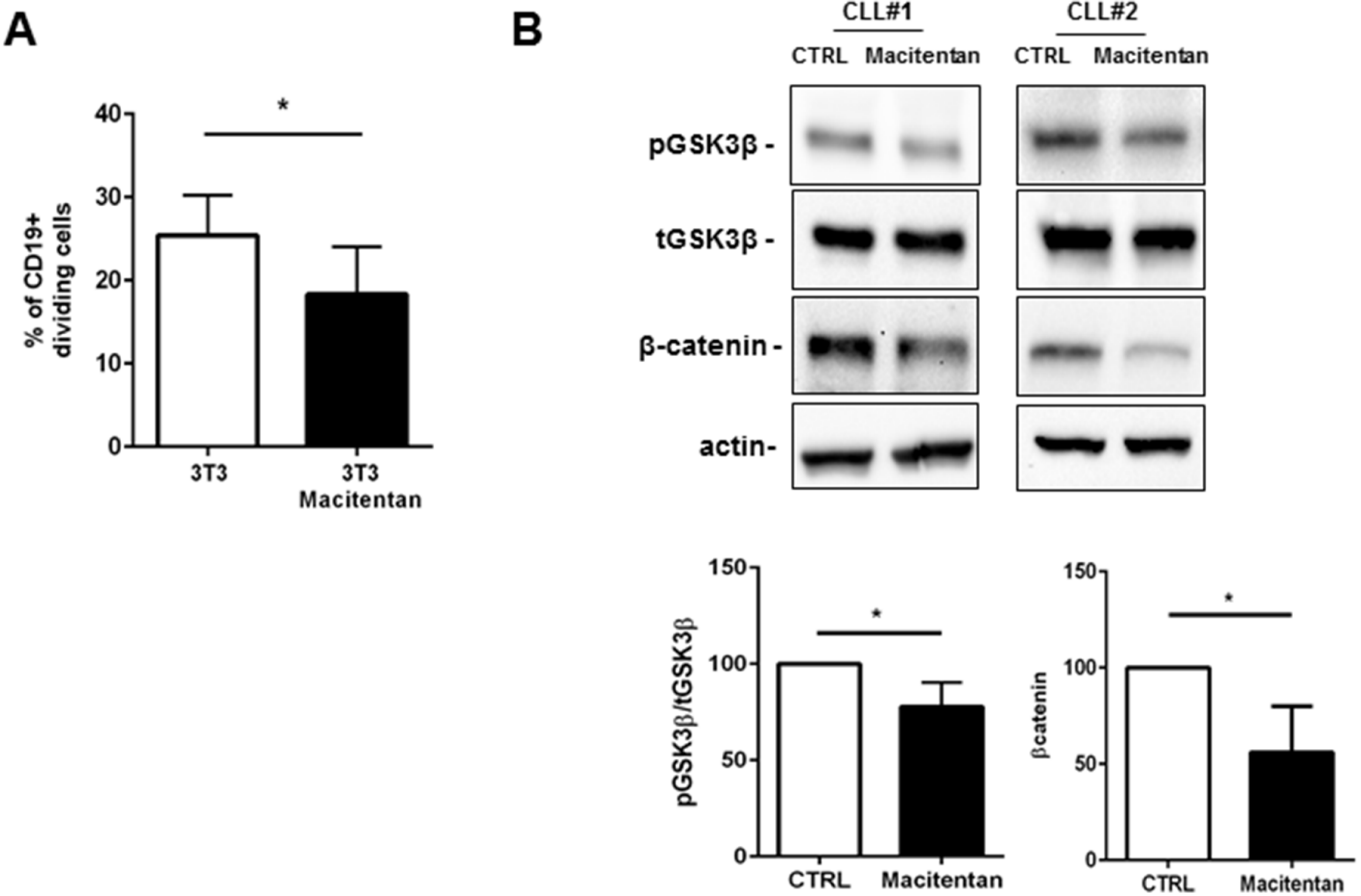
Macitentan affects CLL proliferation mediated by contact with stromal cells **(A)** CFSE-labeled CLL cells were cultured for 4 days alone in complete medium (control) or on 3T3 murine stromal cell layers. Where indicated, CLL cells were incubated for 1 hour with macitentan before co-culture. The proliferative measure was inspected for 4 days, gating the CD19+ live CLL cells. The histograms represent cumulative data at 96 hours of 3 independent experiments by using 3 CLL patients. Data are shown as mean values±SEM of the percentage of dividing CLL cells. The percentage of dividing CLL cells cultured alone was irrelevant. **(B)** CLL cells were either pre-treated or not with macitentan for 1 hour and then cultured for 24 hours. Panel B shows two representative western blots blotted for pGSK3β and β-catenin. Bar diagrams depict densitometric quantification of bands relative to pGSK3β/total GSK3β and β-catenin either in presence or absence of macitentan, normalized on β-actin. Data are presented as mean ± SEM of 4 different CLL samples (^*^P<0.05).

A fraction of CLL cells inside tissues are proliferating, being the cell birth rate 0.1 to 1% of the CLL clone per day. Far from being a marginal event in CLL pathobiology, cell proliferation contributes to clonal evolution of leukemic clone, improvement in disease aggressiveness, relapses after treatments and acquisition of chemoresistance. We asked if interfering with ET-1 signaling through macitentan could counteract the proliferative promptness of CLL cells in contact with stromal cells. As shown in Figure 4A, the percentage of divided CLL cells after 72 hours co-culture in contact with 3T3 cell layer was significantly reduced in the presence of macitentan from 25% to 18%. Due to the established cooperation between ET-1 and β-catenin signaling pathways, we evaluated the ability of macitentan to interfere with β-catenin accumulation in CLL cells. We found that macitentan activates the glycogen synthase kinase 3β (GSK3β) by reducing its phosphorylation at serine 9. Thus, activated β-catenin destruction complex leads to proteasomal degradation of β-catenin, which was detected at lower levels in CLL cells (Figure 4B).

### Macitentan restrains CLL adhesion and movement towards ET-1 and CXCL12

**Figure 5:**
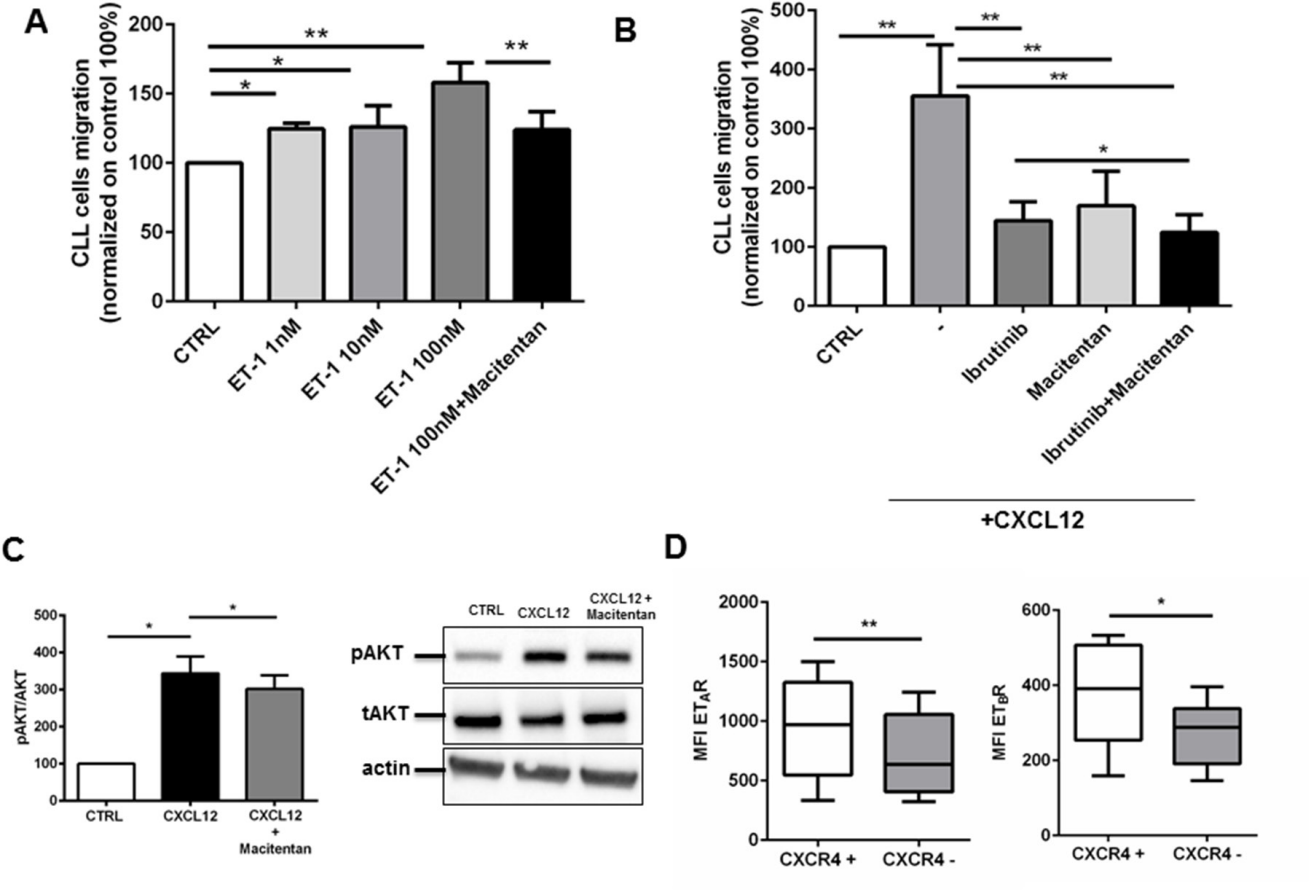
Macitentan affects CLL cell chemotaxis towards ET-1 and CXCL12 **(A)** CLL cells were allowed to migrate towards different doses of ET-1: 1nM, 10nM and 100 nM, or control medium without chemokine. CLL cells were pre-incubated or not with macitentan and migration to 100nM ET-1 was inspected. The bar diagram represents the mean migration (± SEM) of CLL cells from 6 different patients (^*^P<0.05, ^**^P<0.01). **(B)** CLL cells were treated with ibrutinib, macitentan or a combination of both and then allowed to migrate towards CXCL12. The bar diagram represents the mean migration (± SEM) of CLL cells from 8 different patients (^*^P<0.05). **(C)** CD19+ CLL cells isolated from 5 patients were treated with macitentan for 4 hours and then stimulated with CXCL12 for 10 minutes. On the left, bar diagrams represent densitometric quantification of bands relative to pAKT/total AKT normalized on β-actin. Data are presented as mean ± SEM of 5 different CLL samples (^*^P<0.05). On the right, immunoblots show phosphorylation of AKT for one representative CLL sample. **(D)** PBMCs isolated from 8 CLL patients were stained for CD19. CD19+ cells, stained for CXCR4, were divided in positive and negative. For each group, MFI of ET_A_ receptor and ET_B_ receptor was measured. In all experiments, an isotype control sample for each condition was acquired to exclude autofluorescence background. Box blots show the MFI of ET_A_R and ET_B_R in the CXCR4+ and CXCR4- populations (n=8, ^*^P<0.05, ^**^P<0.01).

Homing of CLL cells to the bone marrow and lymph node compartments represents a crucial step in disease progression. We wondered if ET-1 signaling has a role in CLL trafficking. First, we found that CLL cells migrate toward ET-1 in a dose-dependent manner (Figure 5A). Blocking ET receptors on CLL cells by macitentan restrains CLL movement to ET-1 (Figure 5A). Stimulation of CLL cells with ET-1 was able to induce a slight increase of CXCR4 expression (n=5, P<0.05, data not shown). On this line, treatment of CLL cells with macitentan was able to interfere with another crucial chemokine CXCL12, secreted by stromal cells and well known to be essential for CLL migration inside tissues (Figure 5B). Interestingly, macitentan mimics ibrutinib effect on CXCL12-mediated migration, further improving ibrutinib effect when used in combination (from 356±86% for CXCL12-mediated migration to 144±32% for ibrutinib alone and to 124±30% for combination, p=0.032, Figure 5B). We demonstrated that macitentan interferes with CXCR4 signaling pathways. Treating CLL cells with macitentan for 4 hours and then stimulating them with CXCL12 for 10 minutes, phosphorylation of AKT was significantly decreased (Figure 5C, n=4, P<0.05). Accordingly, circulating CLL fraction with higher levels of CXCR4, the CXCL12 receptor, on the cellular surface showed increased density of both ET receptors, likely defining a subpopulation of leukemic cells with more promptness to taste migratory signals (Figure 5D).

**Figure 6:**
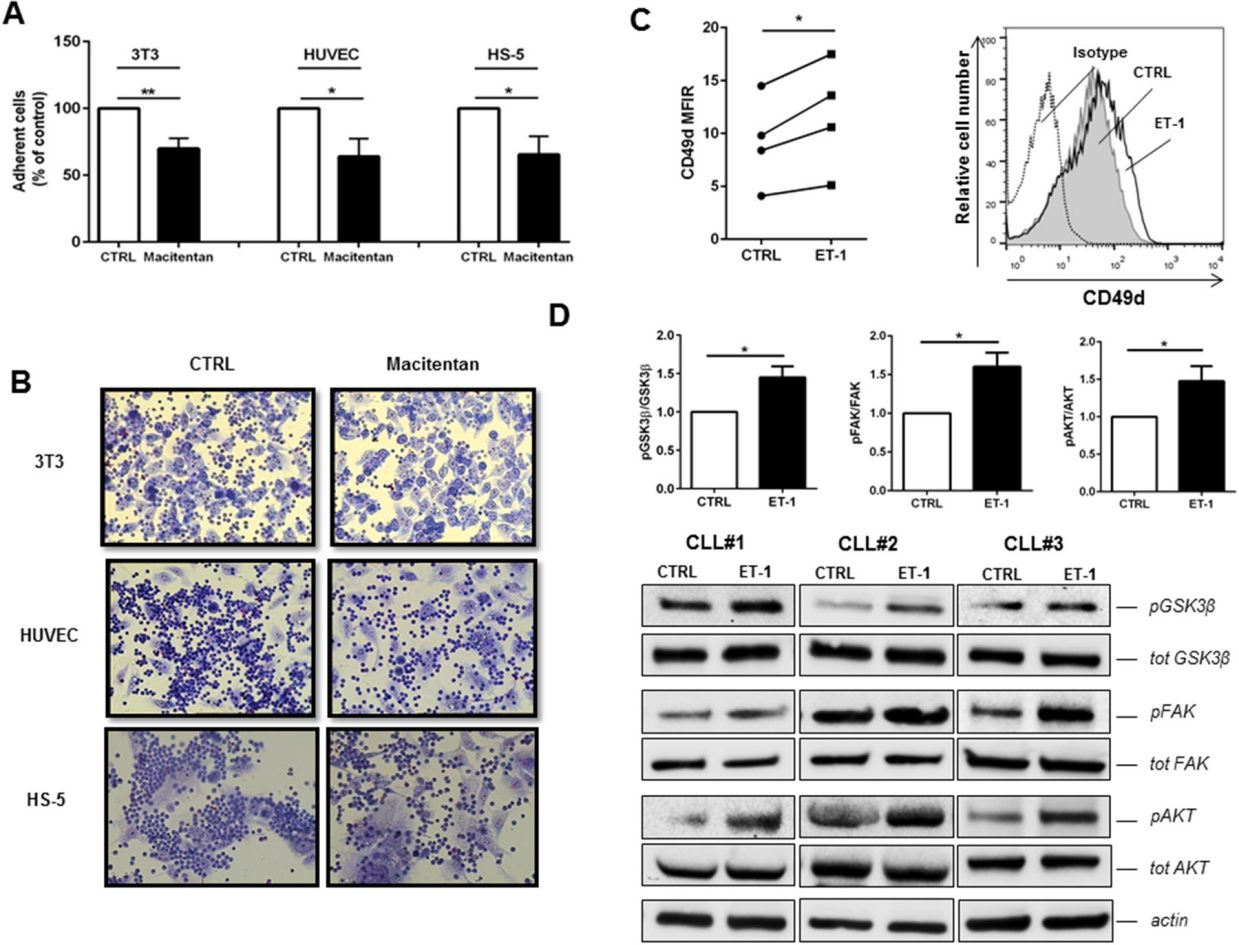
Macitentan inhibits CLL cell adhesion to endothelial and stromal cells **(A)** CLL cells were treated with macitentan and allowed to adhere to 3T3, HUVEC and HS-5 for 4 hours. The bar diagrams represent the mean relative adhesion (±SEM) of CLL cells in the presence of macitentan compared with control (n=6, ^*^P<0.05; ^**^P<0.01). **(B)** Representative phase contrast photomicrographs demonstrating CLL cell adhesion to 3T3, HUVEC and HS-5 either treated or untreated (control) with macitentan. **(C)** CLL cells were stimulated for 24h with ET-1 and then analyzed for CD49d expression. Values of untreated and treated samples are connected by lines (n=4, ^*^P<0.05, ^**^P<0.01). On the right, a representative histogram shows the fluorescence intensity of CD19+ CLL cells after treatment with ET-1 stained with anti-CD49d antibody. **(D)** Bar diagrams depict densitometric quantification of bands relative to pGSK3β/tot GSK3β, pFAK/tot FAK and pAKT/tot AKT ratios either in presence or absence of ET-1 stimulation, normalized on β-actin. Data are presented as mean ± SEM of 5 different CLL samples (^*^P<0.05). On the bottom, immunoblots show pGSK3β, pFAK and pAKT in presence of ET-1 stimulation in 3 representative CLL samples.

We then demonstrated that ET-1 signaling improves the adhesive capacity of CLL cells, as inferred on the basis of the following observations. First, macitentan significantly decreases the extent of CLL cells able to firmly attach to stromal and endothelial cell supports (Figures 6A-6B). In particular, a reduction of 30%, 40% and 35% in CLL cells in contact with 3T3 murine stromal cells, HUVEC endothelial cells and HS-5 human stromal cells was measured. Then, an increase in CD49d (also known as VLA-4) expression was measured when CLL cells were stimulated with ET-1 (Figure 6C). On the contrary, we have not found the modulation of other integrins as CD11a, CD29, CD49c and CD18 after stimulation with ET-1 (p=ns, n=7, data not shown). Accordingly, CLL fraction in peripheral blood with higher density of CD49d showed increased levels of ET receptors (Supplementary Figure 3). Lastly, as shown in Figure 6D, ET-1 stimulation activates intracellular key molecules involved in CLL adhesion, as focal kinases.

All together, these findings depict ET-1 signaling as a CLL migration factor, able to regulate the interactions between leukemic cells and surrounding microenvironment by modulating changes in intracellular adhesive signaling and cell surface adhesive molecules.

### ET receptor engagement promotes VEGF expression in CLL cells

**Figure 7:**
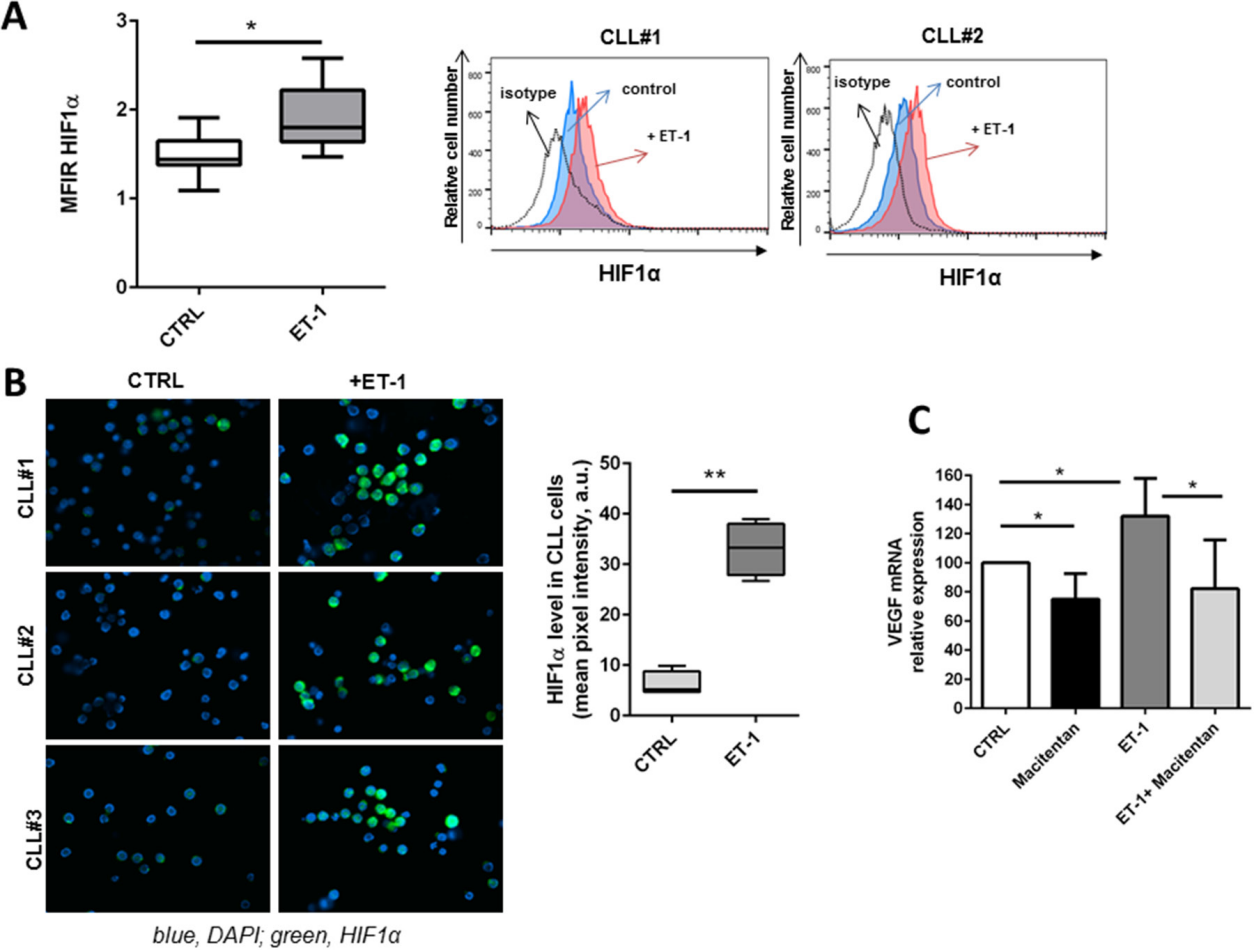
ET-1 stimulation induces a pro-angiogenic profile in CLL cells **(A)** Box plot represents the MFIR of HIF-1α in 7 CLL samples either stimulated or not stimulated with ET-1 for 30 minutes. On the right, histograms show the fluorescence intensity of CD19+ CLL cells after treatment with ET-1 stained with anti-HIF-1α antibody. **(B)** CLL cells were allowed to adhere on the coverslip and then stimulated for 30 minutes with ET-1. HIF-1α induction was analyzed by immunofluorescence microscopy. Three representative CLL samples are shown. On the right, bar diagram represents quantification of cell staining, as mean value obtained from 5 different fields at 400X magnification normalized on control (100%, untreated sample). **(C)** CD19+ CLL cells were stimulated or not with ET-1 either in presence or absence of a pre-treatment with macitentan. Bar diagram depicts VEGF transcriptional levels (n=5, ^*^P<0.05).

ET-1 has been shown to promote angiogenesis by directly controlling EC proliferation and indirectly by inducing vascular endothelial growth factor (VEGF) expression [15]. In CLL, ET-1 signaling promotes HIF-1α accumulation as demonstrated by flow cytometry (Figure 7A) and immunofluorescence (Figure 7B). HIF-1α transcript levels were not affected by ET-1 treatment in CLL cells (data not shown), suggesting that its accumulation may be due to an increased HIF-1α stability as previously reported in melanoma cells [16]. We also detected higher VEGF expression in CLL cells upon exposure to ET-1 (Figure 7C). Treating CLL cells with macitentan reduces their pro-angiogenic profile by interfering with VEGF expression (Figure 7C).

## DISCUSSION

Aberrant activation of ET-1 axis is now recognized as a common mechanism underlying the progression of several solid tumors. Binding two distinct G protein-coupled receptors (GPCRs), ET_A_R and ET_B_R, ET-1 activates a plethora of intracellular signals strictly interconnected with various well-characterized pathways that act in a synergistic and combinatorial manner to favor survival and proliferation, adhesion and migration and to dysregulate the balance of angiogenesis and immune protection [6]. We investigated the role of ET-1 axis in CLL setting evaluating the possibility to interfere with these signals by using macitentan, a novel potent dual ET_A_/ET_B_ receptor antagonist.

We demonstrated that CLL patients experience a progressive big ET-1 increase in plasma during the natural disease progression from MBL condition to CLL diagnosis and further when uncontrolled CLL cell accumulation needs therapeutic intervention. When CLL clone relapses after chemotherapy an additional increase in big ET-1 was detected, whereas disease control exerted by ibrutinib treatment rapidly reduced big ET-1 levels. Higher big ET-1 levels detected in CLL patients at diagnosis predict shorter time to first treatment and characterize CLL clone with adverse prognostic features such as unmutated immunoglobulin genes and deletion 17p and 11q as demonstrated in a previous study [14]. Synthesis of the biologically active ET-1 peptide is a multistep process. The primary translation product of ET-1 gene is the 212-aa prepro ET-1, which is cleaved by an endothelin converting enzyme (ECE-1) to form the 38-aa big ET-1 and then to the biologically active 21-aa ET-1 peptide [4]. We assessed big ET-1 levels instead of the active peptide ET-1 due to the very low levels of ET-1 in plasma and the short half-life (1.5 minutes) [17]. Our results are in line with a well-documented association between circulating big ET-1, adverse prognostic features and shorter survival in patients with various solid tumors [18–21]. Altogether these findings strongly suggest an impact of ET-1 axis in CLL pathobiology and envision the possibility to explore a novel class of agents interfering with ET receptors, also in combinations with new drugs currently available or actually tested in clinical trials such as ibrutinib, idelalisib and ABT-199.

We speculated that disrupting ET-1 signaling pathways may concomitantly affect a panel of aberrant biological functions typical of CLL cells. Resistance to apoptosis is a hallmark of CLL disease and it is induced by intrinsic genetic lesions and extrinsic signals from microenvironmental elements that cooperate to keep active intracellular pro-survival pathways such as PI3K-Akt signaling or up-regulate anti-apoptotic molecules such as BCL-2, BCL-X_L_ and survivin. ET-1 acts as an anti-apoptotic factor in different cell types by modulating PI3K-dependent Akt activation and by increasing pro-survival factors [14, 22–24]. We found that macitentan induces CLL spontaneous apoptosis and hampers the survival advantage acquired by CLL cells both in contact with stromal cells and in response to BCR activation. In CLL, ET-1 signaling converges to BCR intracellular cascade. As consequence, macitentan cooperates with ibrutinib reducing the phosphorylation of crucial tyrosine kinases in BCR signaling, i.e. BTK and ERK.

Moreover, ET-1 induces the expression of anti-apoptotic protein BCL-2, thus contributing to apoptosis resistance in CLL cells. This leads to the possibility that ET-1 signaling may be partially involved into CLL resistance to BCL-2-specific compound ABT-199. Beside the exciting clinical results of venetoclax (ABT-199), it has to be considered that some patients harbor sub-populations of resistant cells that mediate disease recurrence [25, 26]. Venetoclax resistance seems to be mediated by microenvironmental signals that lead to BCL-2, BCL-X_L_ and MCL-1 accumulation in leukemic cells [27–30]. We demonstrated that macitentan affects ET-1-mediated BCL-2 accumulation and further increases ABT-199-mediated apoptosis in CLL cells. Interestingly, a reduction in CLL protection mediated by stromal cells against ABT-199 effect was detected in combination with macitentan. ET-1 signaling has been correlated to acquisition of chemo-resistance in several solid tumors such as ovarian, prostate, colon, cervical carcinoma and osteosarcoma [12, 31–35]. In ovarian cancer, ET-1 confers resistance to paclitaxel-induced apoptosis by a BCL-2-dependent mechanism that can be reversed by the addition of specific ET_A_R antagonist [36]. In mice models, macitentan sensitizes ovarian and breast cancer cells to paclitaxel and cisplatinum and glioblastoma cells to temolozomide [9, 10, 12, 13, 37].

CLL cells shape the architecture of infiltrating tissues generating follicular-like structures known as proliferation centers. Inside proliferation centers, leukemic cells engage close connections with surrounding accessory cells of stromal and immune systems that drive proliferative stimuli. Leukemic cells of each patient had definable and often substantial birth rates, varying from 0.1% to greater than 1.0% of the entire clone per day. Moreover, high levels of CLL proliferation are correlated with worse prognosis [38]. Accordingly, higher circulating Ki-67 index was significantly associated with shorter survival [39]. It turns out that proliferation is not a marginal phenomenon in CLL, but represents a crucial step for disease accumulation and clonal evolution. We demonstrated that macitentan interferes with CLL proliferation driven by stromal cells. ET-1 acts as a mitogenic signal both in normal cells including fibroblasts and EC and in various tumor cells throughout the elevation of intracellular free Ca^2+^, activation of mitogen-activated protein kinases and β-catenin signaling [6]. Macitentan reduces β-catenin accumulation in CLL by decreasing the phosphorylation of GSK3β, and thus activating the destruction complex. The interference with β-catenin accumulation together with the inactivation of MAPK and PI3K signaling pathways may account for the reduction in CLL proliferation mediated by macitentan.

The necessity of CLL cells to localize into tissues in order to establish crucial mutualistic crosstalk with tumor microenvironment represents the Achilles’ heel of leukemic clone. Recirculation of CLL cells from peripheral blood to tissue compartment requires the cooperation between several chemokine receptors and adhesion molecules. In particular, the chemokine receptor CXCR4 is expressed at high levels in CLL cells and mediates CLL cell chemotaxis to CXCL12, migration across vascular endothelium, and then beneath and underneath CXCL12-secreting stromal cells [40]. In addition, the α_4_β_1_ integrin VLA-4 (CD49d) cooperates with chemokine receptors in CLL cell adhesion to stromal cells. We demonstrated that ET-1 acts as a chemotactic and adhesive factor for CLL cells. Indeed, ET-1 promotes CLL migration, favors CD49d expression in CLL cells and mediates intracellular phosphorylation of focal adhesion kinase (FAK). Macitentan restrains CLL movement to both ET-1 and CXCL12, interfering with CXCR4/CXCL12 axis. In addition, macitentan reduces CLL adhesion to endothelial and stromal cells.

Preclinical results indicate good efficacy when macitentan was administered in combination with chemotherapy in solid tumors. Recently, macitentan plus paclitaxel significantly increased overall survival by producing complete responses in all mice tested harboring brain metastasis of breast and lung cancer [37]. In chemoresistant ovarian cancer, tumor suppression and reduction of metastatic progression were reported with co-treatment of macitentan and cisplatinum [12]. In another study, the majority of mice (96%) bearing orthotopically implanted glioblastoma-resistant cancer cells and treated with macitentan and temolozomide had no evidence of disease [9]. Beside these promising preclinical findings in other tumor settings, the use of dual ET_A_R and ET_B_R antagonists could offer the advantage in CLL to target not only tumor cells (which express both receptors) but also surrounding EC, stromal cells and immune cells, which all expressed ET_B_R. Indeed, macitentan induces apoptosis in tumor-associated endothelial cells [10, 13, 37]. Moreover, ET-1 acts on cancer-associated fibroblasts to promote the formation of supporting stroma [41] and favors macrophage migration and production of inflammatory mediators, while inhibits T cell homing to tumors [42]. Further studies are necessary to evaluate the effects of macitentan treatment on immune elements in CLL patients.

In conclusion, our findings demonstrate that macitentan impairs the pleiotropic actions of ET-1 signaling, concomitantly affecting multiple aberrant functions of CLL cells, i.e. survival, proliferation, angiogenesis, migration and chemoresistance. The possibility to interfere with multiple signals directly involved in CLL maintenance and progression and also with the mutualistic dialogue between CLL and microenvironmental accessory cells by using macitentan, also testing it in combination strategies with novel therapeutic agents, seems attracting and needs further investigation in CLL.

## MATERIALS AND METHODS

### Patients and samples

Blood samples from CLL patients were obtained from the Hematology Unit of Modena Hospital in Italy with a protocol approved by the Institutional Review Board. All patients tested in *in vitro* experiments were untreated at blood collection, whereas CLL patients relapsed from 1 or multiple treatments or treated with ibrutinib were included in the evaluation of big ET-1 plasma levels. All patients provided written informed consent in accordance with the declaration of Helsinki. Peripheral blood mononuclear cells (PBMCs) were isolated by density gradient centrifugation and used fresh or cryopreserved in liquid nitrogen. Plasma was obtained centrifuging peripheral blood for 15 minutes at 2000 rpm and then stored at -80°C. To purify CLL, PBMCs were incubated with CD19-specific Microbeads (Miltenyi Biotec, Bergisch Gladbach, Germany) and separated by AutoMACS (Miltenyi Biotec), obtaining a purity >99% as assessed by flow-cytometry. Macitentan and ibrutinib were purchased by Selleckchem (Munich, Germany) and dissolved in DMSO, used as vehicle. ET-1 peptide (H-6995) was obtained by Bachem (Bubendorf, Switzerland) and solubilized in 5% acetic acid, used as vehicle in all experiments.

### Migration assays

To test migration, CLL cells were pre-treated with 1 μM macitentan and/or 0.5 μM ibrutinib or vehicle for 1 hour. ET-1 at increasing doses of 1 nM, 10 nM and 100 nM or CXCL12 (also known as stromal-cell derived factor-1, SDF-1α) (Peprotech, Rocky Hill, NJ, USA) at 200 ng/mL were used as chemoattractant and placed in RPMI+0.2% BSA (600 μL) in the bottom compartment of Millicell Cell Culture Inserts (Millipore, Billerica, USA). Then, 200 μL of cell suspension (3×10^5^ cells) were added to the upper inserts. The chambers were incubated at 37°C in humidified air with 5% CO_2_ for 24 hours. Migrated cells were labeled with 4 μM Calcein-AM (Sigma-Aldrich, St. Luis, MO, USA) for 45 min and quantified by fluorescence plate reader Infinite200 (Tecan, Männedorf, Switzerland) at an excitation wavelength of 485 nm and an emission of 520 nm.

### Adhesion assays

CLL cells were added onto the confluent layers of Human Umbilical Vein Endothelial Cells (HUVEC) or 3T3 murine stromal cells or HS-5 human stromal cells and allowed to adhere for 4 hours at 37°C. Prior to incubation, CLL were treated with 1 μM macitentan or vehicle for 1 hour. Then, CLL firmly adherent to HUVEC, 3T3 and HS-5 layer were counted by staining with APC-conjugated anti-CD19 antibody (Miltenyi Biotec) as previously described [43].

### Viability assays

CLL viability was inspected in three experimental settings. First, cells were incubated or not with 1 μM macitentan or DMSO for 1 hour at 37°C following stimulation with 100 nM ET-1 or vehicle for 24 hours. In a second setting, CLL cells were incubated for 1 hour at 37°C with macitentan or DMSO then plating cells onto confluent 3T3 murine stromal cells and assessing CLL viability until 96 hours. 3T3 cells were excluded using a morphological gate, as their relative size and granularity (forward scatter and side scatter) is clearly distinguishable from that of lymphocytes. In some experiments, ABT-199 was added at a dose of 1 nM in CLL cells cultured alone and 1μM in CLL cells co-cultured with 3T3. In a third setting of experiment, CLL cells were pre-incubated with 1 μM macitentan and/or 0.5μM ibrutinib or vehicle for 1 hour at 37°C, then stimulating or not cells with 10 μg/mL goat F(AB’)2 fragment to human IgM (5FCμ) (MP Biomedicals, Santa Ana, CA, USA) and measuring CLL viability until 96 hours. Apoptotic cell death was analyzed using Annexin V-FITC and Propidium Iodide (PI) staining (eBioscience, San Diego, CA, USA). Events were acquired using a FACSCalibur cytometer (Becton Dickinson) and then analyzed by FlowJo Software (Tree Star, Ashland, OR, USA). The combination between ibrutinib and macitentan or ABT-199 and macitentan was defined synergistic according to the multiplicative model proposed by Larsson R et al [44].

### Immunoblotting

Purified CLL cells were exposed to 100 nM ET-1 for 30 minutes. In another experiment, CLL cells were pre-incubated with 1 μM macitentan and/or 0.5μM ibrutinib or vehicles for 1 hour at 37°C, then stimulating or not cells with 10 μg/mL goat F(AB’)2 fragment to human IgM (5FCμ) for 10 minutes. Cells were then lysed on ice for 10 minutes with lysis buffer supplemented with dithiothreitol and protease inhibitor cocktail (BioVision, Milpitas, CA, USA). Proteins (80μg/lane) were electrophoresed on 4% to 20% SDS-polyacrylamide gradient gels (Biorad laboratories, Hercules, CA, USA). Membranes were immunoblotted with primary antibodies listed in Supplementary Table 1. Then, membranes were incubated with species-specific horseradish peroxidase (HRP)-conjugated secondary antibody (diluted 1:50000; GE Healthcare, Uppsala, Sweden) for 1 hour and developed using HRP conjugates WesternBright Sirius (Advansta, Menlo Park, CA, USA). Images were acquired and analyzed using Image Lab Software v.3.0 (Biorad Laboratories).

### Immunofluorescence

HIF-1α expression in CD19+ CLL cells was evaluated by an intracytoplasmatic immunofluorescence staining. CLL cells were plated in RPMI+10% FBS on coverslip in a 24-well plate and let them to adhere overnight. The day after, cells were stimulated with 100 nM ET-1 or vehicle. After 4 hours, cells were fixed. After washes, anti-HIF-1α antibody (Novus Biologicals) was loaded on coverslips and incubated overnight at +4°C. The next day, the coverslips were washed and incubated with Alexa-Fluor 488 conjugated secondary antibody (Life Technologies). The immunofluorescent images were visualized with Leica DMRA2 fluorescence microscopy (Leica Microsystems) equipped with a DC350 FX camera. Pixel intensity analyses were performed using the ImageJ (downloadable at http://rsbweb.nih.gov/ij/) software. Mean pixel intensity was calculated by defining a region of interest (ROI) and measuring green fluorescence pixel intensity. Results are expressed as fold change compared to untreated control.

### Enzyme-linked immunosorbent assays

Big ET-1 levels in conditioned media and plasma samples were measured using big Endothelin-1 (human) EIA kit (Enzo Life Sciences, Farmingdale, NY, USA). The mean minimum detectable dose was 0.23 pg/mL. Each sample was tested in duplicate and concentrations were reported in pg/mL.

### Quantitative PCR

RNA was extracted with the RNeasy Plus Mini kit (Qiagen, Valencia, CA, USA) and reverse transcribed using SS VILO Mastermix (Life Technologies). Ten nanograms per reaction of cDNA were analyzed in Real-Time PCR on LightCycler 480 v.2 (Roche) using SYBR Green Master Mix (Applied Biosystems) and specific primers designed for ET-1 and VEGF or the internal control Glyceraldehyde 3-phosphate dehydrogenase (GAPDH) gene. All samples were run in duplicate. Comparative relative expression was calculated using the delta Ct method and normalized to a calibrator sample (Universal Human Reference RNA; Stratagene, Cedar Creek, TX). To exclude non-specific amplification and primer-dimer formation, a dissociation curve analysis was performed and PCR products were tested by agarose gel electrophoresis.

### Flow cytometry

PBMCs from CLL patients were incubated with APC-conjugated anti-CD19 antibody (Becton Dickinson, San José, CA, USA), PE-conjugate anti-CD184 (CXCR4) or PE-conjugate anti-CD49d (Miltenyi Biotec) and rabbit ET_B_R (N-terminal) antibody (Sigma-Aldrich, St. Luis, MO, USA) or rabbit polyclonal ET_A_R antibody (Abgent, S.Diego, CA, USA) for 30 min in ice followed by FITC-conjugated Goat anti-rabbit Ig for 30 min in ice (Becton Dickinson). For each sample an isotype control was prepared in parallel. In other experiments, CLL cells were incubated for 30 minutes with 100 nM ET-1 or vehicle (control sample), then stained with the following antibodies and corresponding isotype controls: anti-HIF-1α antibody (Novus Biologicals, Abingdon, UK) followed by FITC-conjugated goat anti-rabbit staining.

### Proliferation assay

CFSE (5-[and 6]-Carboxyfluorescein diacetate succinimidyl ester; eBioscience) dilution assay was used to trace cell division by flow cytometry. CD19+ CLL cells, stained with CFSE, were incubated or not with macitentan and then plated onto the confluent 3T40L layer. The proliferative measure was evaluated after 4 days, gating the CD19+ alive CLL cells, and analyzed using Modfit software. For the analysis, every generation of cells appears as a different peak on a flow cytometry histogram.

### Statistical analyses

Data were analyzed using SPSS version 20.0 (SPSS, Chicago, IL, USA). In some experiments, results were normalized on control (100%) (vehicle-treated samples). Normalization was performed by dividing the value of a particular treated sample to the value of the corresponding sample treated with vehicle. *P* values were calculated by Student t test (^*^P<0.05, ^**^P<0.01). Data are presented as mean and standard error of the mean (SEM) is depicted as error bars.

## SUPPLEMENTARY MATERIALS FIGURES AND TABLES



## Abbreviations

CLL: Chronic Lymphocytic Leukemia
ET-1: endothelin-1
ET_A_R: endothelin receptor type A
ETBR: endothelin receptor type B
BCR: B-cell receptor
HIF-1α: hypoxia-inducible factor-1
VEGF: vascular endothelial growth factor
EC: endothelial cells
MBL: monoclonal B-cell lymphocytosis
WBC: white blood cell
BTK: Bruton tyrosine kinase
GSK3β: glycogen synthase kinase 3β
PI3K: phosphatidylinositol 3-kinase
MAPK: mitogen-activated protein kinase
FAK: focal adhesion kinase
SDF-1α: stromal-cell derived factor 1
HUVEC: human umbilical vein endothelial cells
HS-5: human stromal cells 5
MFIR: mean fluorescence intensity.
