# Supplementary material for: Macitentan, a double antagonist of endothelin receptors, efficiently impairs migration and microenvironmental survival signals in chronic lymphocytic leukemia
Source: Oncotarget. 2017 Sep 27;8(52):90013–27. doi: 10.18632/oncotarget.21341 (PMC5685728; doi:10.18632/oncotarget.21341)
Supplement: Supplementary file 1 [file oncotarget-08-90013-s001.pdf]

Macitentan, a double antagonist of endothelin receptors, efficiently impairs migration and microenvironmental survival signals in chronic lymphocytic leukemia

SUPPLEMENTARY MATERIALS

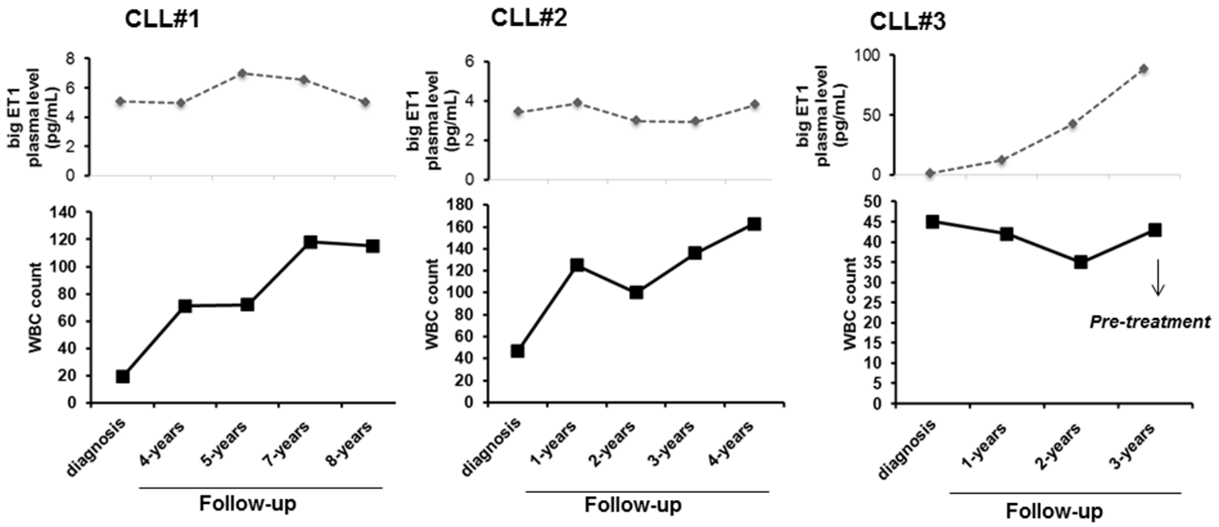

Supplementary Figure 1: Big ET-1 plasma levels do not correlate with white blood cell (WBC) count. Three representative CLL patients are depicted, showing WBC count (x10^9/L) at diagnosis and during follow-up (bottom panels) and big ET-1 plasma levels in pg/mL (upper panels). X-axis represent years from diagnosis in both upper and bottom panels.

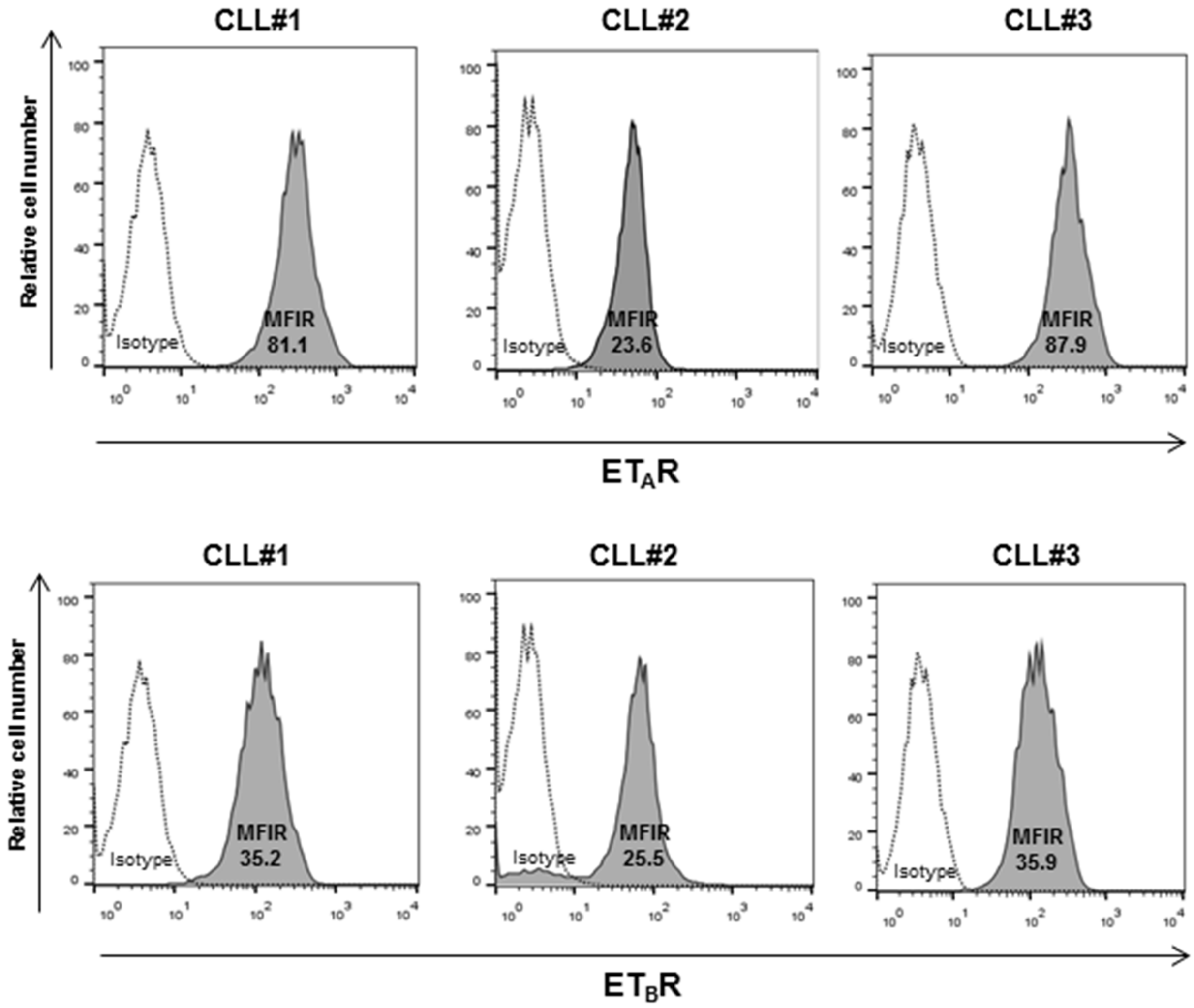

Supplementary Figure 2: CLL cells express ET_A and ET_B receptors. Displayed are flow cytometric histograms depicting the relative fluorescence intensity of 3 CLL representative CLL samples stained with anti-CD19 and anti-ET_AR (upper panel) and anti-ET_BR Abs (bottom panel). Mean fluorescence intensity ratio (MFIR) is displayed above the histograms and is calculated by dividing the mean fluorescence intensity for ET_AR and ET_BR by the mean fluorescence of the corresponding isotype control.

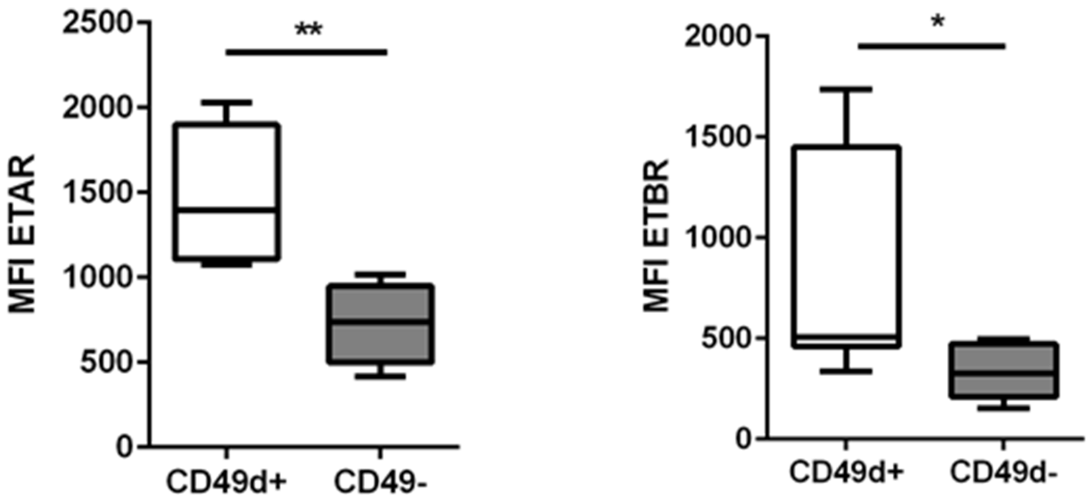

Supplementary Figure 3: Differential expression of ET_AR and ET_BR in CD49+ and CD49- CLL populations. PBMCs isolated from 8 CLL patients were stained for CD19. CD19+ cells, stained for CD49d, were divided in positive and negative. For each group MFI of ET_A receptor and ET_B receptor were measured. In all the experiments, an isotype control sample for each condition was acquired to exclude autofluorescence background. Box blots show the MFI of ET_AR and ET_BR in the CD49d+ and CD49d- populations (n=8, $^{*}P<0.05$, $^{**}P<0.01$).

Supplementary Table 1: Antibodies used in immunoblotting

Antibody	Manufacturer
Anti-phospho-BTK Tyr^{223} rabbit	Cell Signaling Tech, Beverly, MA
Anti-total BTK rabbit	Cell Signaling Tech, Beverly, MA
Anti-phospho-Erk 1/2 rabbit	Cell Signaling Tech, Beverly, MA
Anti-Erk 1/2 rabbit	Cell Signaling Tech, Beverly, MA
Anti-phospho-Akt rabbit	Cell Signaling Tech, Beverly, MA
Anti-Akt rabbit	Cell Signaling Tech, Beverly, MA
Anti-phospho-GSK3β (Ser9) rabbit	Cell Signaling Tech, Beverly, MA
Anti-GSK3β	Cell Signaling Tech, Beverly, MA
Anti-phospho-FAK (Tyr925) rabbit	Cell Signaling Tech, Beverly, MA
Anti-FAK rabbit	Cell Signaling Tech, Beverly, MA
Anti-βcatenin rabbit	Cell Signaling Tech, Beverly, MA
Anti-Bcl2 mouse	Cell Signaling Tech, Beverly, MA
Anti-CD19 rabbit	Cell Signaling Tech, Beverly, MA
Anti-βactin mouse	Abcam, Cambridge, UK
